# Bioclimatic conditions of the Lower Silesia region (South-West Poland) from 1966 to 2017

**DOI:** 10.1007/s00484-020-01970-5

**Published:** 2020-10-01

**Authors:** Arkadiusz Głogowski, Krystyna Bryś, Paolo Perona

**Affiliations:** 1 Institute of Environmental Protection and Development, Wrocław University of Environmental and Life Sciences, ul. C. K. Norwida 25, 50-375 Wrocław, Poland; 2 School of Engineering, The University of Edinburgh, Mayfield Road EH93JL Edinburgh, Scotland, UK

**Keywords:** UTCI, Biometeorology, Forecasting, ARMA model

## Abstract

This work analyses the temporal and spatial characteristics of bioclimatic conditions in the Lower Silesia region. The daily time values (12UTC) of meteorological variables in the period 1966–2017 from seven synoptic stations of the Institute of Meteorology and Water Management (IMGW) (Jelenia Góra, Kłodzko, Legnica, Leszno, Wrocław, Opole, Śnieżka) were used as the basic data to assess the thermal stress index UTCI (Universal Thermal Climate Index). The UTCI can be interpreted by ten different thermal classes, representing the bulk of these bioclimatic conditions. Stochastic autoregressive moving-average modelling (ARMA) was used for the statistical analysis and modelling of the UTCI as well as separately for all meteorological components. This made it possible to test differences in predicting UTCI as a full index or reconstructing it from single meteorological variables. The results show an annual and seasonal variability of UTCI for the Lower Silesia region. Strong significant spatial correlations in UTCI were also found in all stations of the region. “No thermal stress” is the most commonly occurring thermal class in this region (about 38%). Thermal conditions related to cold stress classes occurred more frequently (all cold classes at about 47%) than those of heat stress classes (all heat classes at about 15%). Over the available 52-year period, the occurrence of “extreme heat stress” conditions was not detected. Autoregressive analysis, although successful in predicting UTCI, was nonetheless unsuccessful in reconstructing the wind speed, which showed a persistent temporal correlation possibly due to its vectorial origin. We conclude thereby that reconstructing UTCI using linear autoregressive methods is more suitable when working directly on the UTCI as a whole rather than reconstructing it from single variables.

**Electronic supplementary material:**

The online version of this article (10.1007/s00484-020-01970-5) contains supplementary material, which is available to authorized users.

## Introduction

Bioclimatology finds applications in many fields such as climate change (Wu et al. 2019), health research (Bröde et al. 2018), epidemiology (Di Napoli et al. 2018), military (Galan and Guedes 2019), and urban planning or even to determine the attractiveness of tourist places such as coastal towns or health resorts (Błażejczyk and Kunert 2011b; Ge et al. 2017). However, at a time when meteorological weather forecasts can be modelled anywhere on the planet, there are still many locations that do not possess historical records of meteorological measurements. Forecast for these regions, often including cities or attractive areas, therefore relies purely on extrapolation from the modelled data. The Lower Silesia region accounts for about 20% of all health resorts in Poland to which tourists and the sick go in order to recover or improve their health conditions. Despite the fact that only a few of those health resorts have historical records of meteorological measurements, all resorts are required to periodically report on the biometeorological conditions in the region so as to obtain standard certifications.

Over the last century, scientists have proposed many different bioclimatic indicators. In a review of these bioclimatic indicators, de Freitas and Grigorieva (2017) showed that 165 indicators may be gathered into eight groups depending on the type and nature of these indicators. The Universal Thermal Climate Index (UTCI) was placed within the group representing an energy balance stress index. Its multivariate component definition makes it a versatile bulk index for representing comprehensive bioclimatic conditions (de Freitas and Grigorieva 2017). The World Meteorological Organization (WMO) officially promoted the use of UTCI as the most suitable tool for determining bioclimatic conditions at the international symposium in April of 2009 (WMO 2009). In the last decade, Polish scientists have used UTCI in different parts of Poland and they confirmed that this index is well suited for describing the local bioclimatic conditions (Błażejczyk et al. 2010, 2012, 2013; Chabior 2011; Kuchcik et al.^2013^; Okoniewska and Więcław 2013; Nidzgorska-Lencewicz 2015; Bryś and Ojrzyńska 2016; Rozbicka and Rozbicki 2016, 2018). UTCI has also been used worldwide. In Europe, for example, Novak (2013) investigated its use for regions of the Czech Republic. From the perspective of tourism, researchers have analysed changes of UTCI in Greece, Luxemburg, and Hungary (Matzarakis and Nastos 2011, 2013; Nemeth^2011^). Outside Europe, Bröde et al. (2012b) have made predictions using UTCI in South Brazil, Coutts et al. (2016) in Australia, Maciejczyk et al. (2017) in the Arctic, and Ndetto and Matzarakis (2015) in Tanzania.

In this paper the UTCI was used to determine the biometeorological conditions of the Lower Silesia Voivodeship (south-west Poland). We present a broad statistical analysis of the bioclimatic conditions in terms of the UTCI, and use a stochastic modelling approach to test its reconstruction either as a whole or starting from single meteorological variables that define it. We show how statistics of biometeorological conditions are related to the available synoptic stations in the Lower Silesia region. The significantly strong statistical spatial relationships seem to support the modelling of bioclimatic conditions for those locations that do not have a history of meteorological measurements. However, the stochastic analysis appears to be suitable for modelling the UTCI directly rather than starting from its meteorological components.

## Materials and methods

### Location and available data

**Fig. 1:**
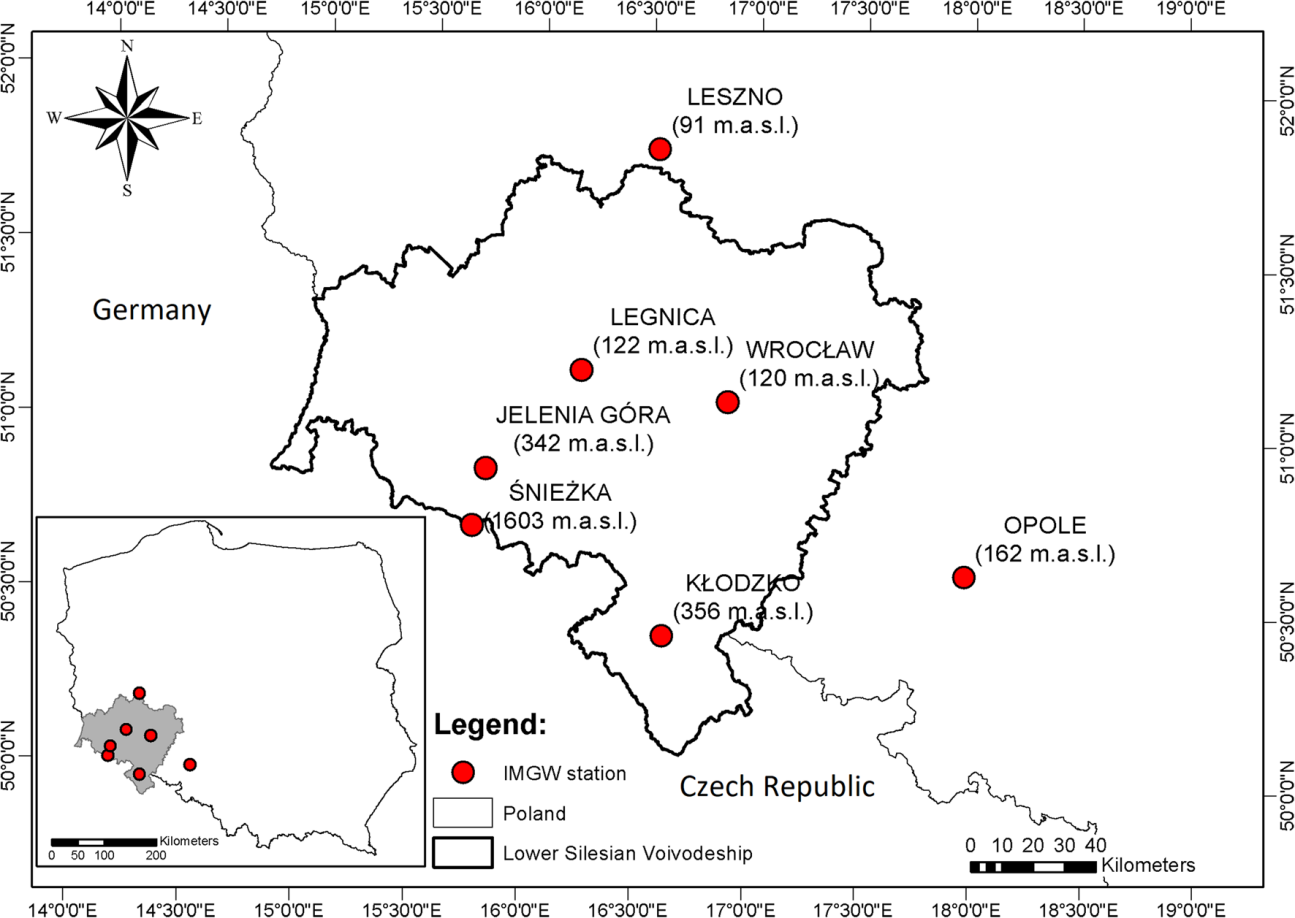
Location of Institute of Meteorology and Water Management (IMGW) stations in Lower Silesia (SW Poland) with heights of the stations in metres above sea level (m a.s.l)

The meteorological data for seven synoptic stations (Fig. 1) used in the article have been provided by the Institute of Meteorology and Water Management https://dane.imgw.pl/ by way of the climate R package (Czernecki et al. 2020). Air temperature, water vapour pressure, wind speed, and cloud cover for the years 1966–2017 were used. For the calculation 1200 UTC observations (in total 132,951 observations;18,993 per station) were used (Błażejczyk and Kunert 2011b). All analysed synoptic stations are part of the WMO database and they fulfil WMO standards (Jarraud 2008) including measurements of the wind speed at a height of 10 m above ground level. Leszno and Legnica stations had gaps of measurements in cloud cover for the years 1966–1977 (about 12% of data for these stations). For the UTCI, calculations gaps for the cloudiness were approximated from the sun’s duration (Bryś et al. 2019).

### Universal Thermal Climate index

The UTCI is a function of air temperature, *T*_*a*_ (^∘^C); wind speed, *v* (m ⋅ s^− 1^); water vapour pressure, *e* (hPa); and mean radiant temperature, *T*_*mrt*_(^∘^C) (Błażejczyk et al. 2013):

1 $$ UTCI = f(Ta,v,e,T_{mrt}). $$

The mean radiant temperature was calculated with the Bioklima software (Błażejczyk 1996), which uses the following mathematical relationships:

2 $$ T_{mrt} = \left( \frac{\frac{R}{Irc}+0.5L_{g}+0.5L_{a}}{s_{h}\cdot\sigma}\right)^{0.25}-273 $$

where *R* is the absorbed solar radiation by the human body (W ⋅ m^− 2^), *Irc* is the coefficient reducing convective and radiative heat transfer through clothing, *L*_*g*_ is the ground radiation (W ⋅ m^− 2^), *L*_*a*_ is the atmosphere’s back radiation (W ⋅ m^− 2^), *s*_*h*_ is the emissivity coefficient for humans (0.95), and *σ* is the Stefan-Boltzmann constant (5.667⋅ 10^− 8^ W⋅ m^− 2^ ⋅ K^− 4^). The absorbed solar radiation (*R*) was calculated using the SolAlt model based on cloudiness (*N* [%]) and position of the sun (hSl [^∘^]); detailed formulas have been reported by Błażejczyk (2005).

The range of limiting conditions in order for UTCI to be applicable are as follows: wind speed between 0.5 and 20 m ⋅ s^− 1^ (Novak 2013), air temperature from − 50 to 50 ^∘^C, mean radiant temperature and air temperature difference *T*_*mrt*_ − *T*_*a*_ from − 30 to 70 ^∘^C, and relative humidity that needs to be higher than 5% (Bröde et al. 2012a). In the analysed period, only the wind speed exceeded the prescribed limiting conditions. About 2% (i.e. 2768 obs.) of the data were greater than 20 m ⋅ s^− 1^ and about 4% (i.e. 5420 obs.) of the data were lower than 0.5 m ⋅ s^− 1^. When wind data exceeded the upper limiting conditions, the UTCI reached its utmost limits. Therefore, following Novak (2013), it was decided to cut off wind data at the limiting conditions and include them in the analysis. The calculated UTCI values correspond to the conditions of a man walking at a speed of 4 km/h (which is equivalent to metabolic changes of 2.3 MET). Activities like mountain hiking will represent a higher metabolic rate; thus, people would have a slightly higher perception than the UTCI prediction.

The scale of thermal stress classes was taken from the physiological model proposed by Bröde et al. (2012a), which is shown in Table 1.

**Table 1:** Assessment scale of UTCI (Havenith et al. 2012)

UTCI ^∘^C	Heat stress classes	Number
UTCI> 46	Extreme heat stress	10
46>UTCI> 38	Very strong heat stress	9
38>UTCI> 32	Strong heat stress	8
32>UTCI> 26	Moderate heat stress	7
26>UTCI> 9	No thermal stress	6
9>UTCI> 0	Slight cold stress	5
0>UTCI>-13	Moderate cold stress	4
− 13>UTCI>-26	Strong cold stress	3
− 26>UTCI>-40	Very strong cold stress	2
− 40>UTCI	Extreme cold stress	1

### ARMA model

Several works in the literature have reported on the significant relationships between UTCI and various climatic oscillations (Owczarek 2019). For example, there is a significant link between atmospheric circulations (e.g. North Atlantic Oscillation, NAO) and climatic conditions in Poland (Marsz et al. 2019). Since climatic oscillations have successfully been modelled by means of stochastic models, we propose to use AutoRegressive Moving Average models (Maidment et al 1993) to decompose bioclimatic conditions (according to UTCI) into their fundamental linear components (i.e. temporal correlation and noise).

AutoRegressive Moving Average (ARMA) models (3) have widely been used in hydrology (Salas et al. 1985; Haltiner and Salas 1988; Maidment et al 1993). ARMA(p,q) stands for Auto-Regressive, AR(p) and Moving Average, MA(q). Consider a time series of *N* equally spaced observed data (e.g. temperature) *y*_*t*_, with sample average *μ* = *E*(*y*), statistically stationary (i.e. showing no temporal trends), and temporally correlated. An ARMA(p,q) model may be written to represent such series as:

3 $$ Y_{t} = \mu + \sum\limits_{j=1}^{p} \phi_{j} (y_{t-j}-\mu) +\epsilon+ \sum\limits_{i=1}^{q} \theta_{i} \epsilon_{t-j} $$

with *p* autoregressive parameters *ϕ*(1),...,*ϕ*(*p*) and *q* moving average parameters, *𝜃*(1),....,*𝜃*(*p*). The noise *𝜖*_*t*_ in Eq. 3 is an uncorrelated Gaussian process with zero mean and unit variance (Maidment et al 1993). Alternatively, the time series can first be detrended, and then standardised by subtracting the mean and then divided by the standard deviation. The standardised data with the removed trend can then be checked for the most suitable ARMA model by computing the sample autocorrelation function (ACF) and the sample partial autocorrelation function (PACF) (Brockwell et al. 1991). The sample ACF is defined as :

4 $$ R(t,s) = \frac{E[(Y_{t}-\mu)(Y_{s}-\mu)]}{\sigma^{2}}, $$

where *t* and *s* are indexing the time series and *k* = *t* − *s* is the lag at which the correlation is calculated.

The PACF is defined by the equation :

5 $$ \alpha(k)= corr(Y_{t+1}-E_{y_{1},y_{n}}(Y_{t+1}),Y_{1}-E_{y_{1},y_{n}}(Y_{1})) $$

where:

$E_{y_{1},y_{n}}(Y)$ is best mean square predictor of Y.

If, for example, ACF (4) is nonzero only at lag zero and PACF (5) tails off at lag 2, then the most suitable model is ARMA(0,2), which is coincident with an MA(2). On the other hand, if PACF is nonzero only at lag 0 and ACF tails off at lag, 1 for example, then the best suitable model is ARMA(1,0), which is AR(1). Nonzero ACF and PACF up to a certain lag will otherwise define an ARMA (p,q) model. The purpose of having fitted a suitable ARMA model is to use it in order to decompose the standardised signal into its correlated and uncorrelated components (Brockwell and Davis 2016). Similarly, the ARMA model can then be used for generating a statistically equivalent time series as well for forecasting. The goodness of the model can be examined for example by using the Akaike Information Criteria (AIC) (Akaike 1974), or better still by the modified AIC called AICc which also accounts for overfitting and overparameterisation (Brockwell et al. 1991). AICc is defined as:

6 $$ AICC(p,q)=Nln(\overline{\sigma^{2}}) + \frac{2(p+q+1)N}{(N-p-q-2)}, $$

where: $\overline {\sigma ^{2}}$ is the maximum likelihood estimator of the noise variance, *N* is the number of observations, and *p*,*q* are the autoregressive and moving average ARMA parameters, respectively.

For each meteorological component (air temperature, vapour pressure, cloudiness, and wind speed) and the computed UTCI from the daily data, we divided the entire record into homogeneous periods showing an almost linear trend, which could then be easily removed. The next step in the decomposition process was to remove the seasonality by subtracting the monthly mean as computed by averaging out the daily data within the same month. Data were then standardised and the most ARMA(p,q) were estimated using the R software (Team et al 2013). The estimated autoregressive model was then used for generating new data.

## Results

### Annual and seasonal variability of UTCI in Lower Silesia for the years 1966–2017

Six out of the seven presented stations show comparable biometeorological conditions. The UTCI annual average values are the highest in Opole in 70% of the times for the analysed period (1966–2017) (Fig. 4).

**Fig. 2:**
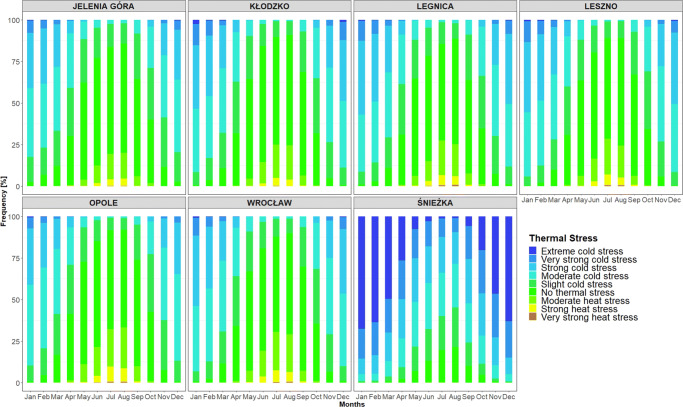
Monthly occurrence of thermal classes for the period 1966–2017 in Lower Silesia

**Fig. 3:**
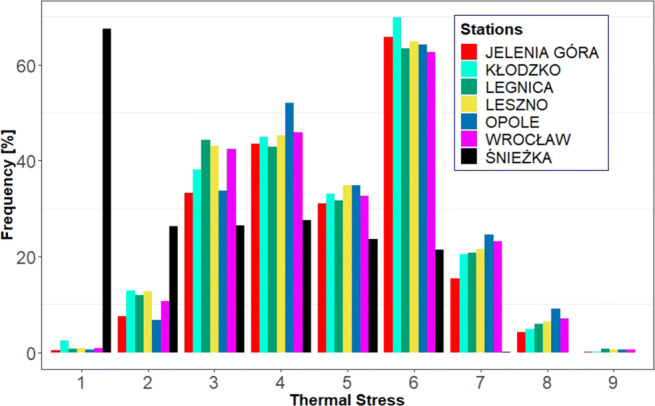
Yearly percentage occurrence of thermal classes for the years 1966–2017 in Lower Silesia

**Fig. 4:**
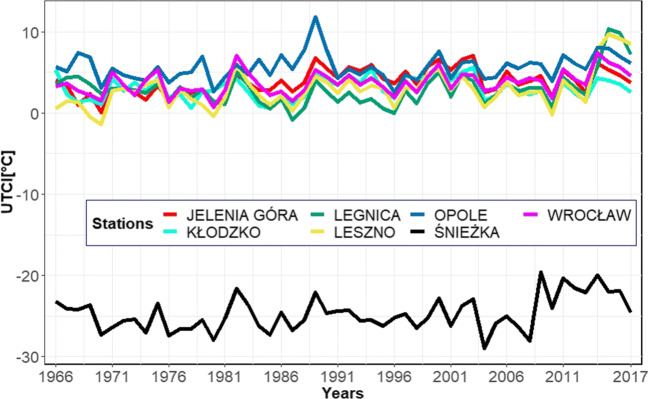
Annual average values of UTCI for all 7 stations in Lower Silesia for the years 1966–2017

UTCI values oscillate for the seven stations almost synchronously, which reflects the high Pearson correlation coefficients computed between all stations (Table 2). The high correlation coefficients confirm the comparability of UTCI oscillations in the studied region. Table 3 shows that UTCI values can become extreme (i.e. according to the classification given in Table 1), reaching high values during winter and low values during summer. However, apart from Śnieżka station, the occurrence of such extremes is generically very low (see Fig. 3). Additionally, the monthly picture offered by Fig. 2 for all stations confirms that there are only few values representing cold stress in summer and heat stress in winter. The case of Śnieżka station needs some further discussion which will be had in the next section. Here, it suffices to say that the frequency and yearly distribution of the frequent extreme UTCI conditions is probably ascribable to its geographical location, elevation, and aspect ratio (Figs. 3 and 4).

**Table 2:** Pearson test correlation of 1200 UTCI values between seven analysed stations

	Kłodzko	Legnica	Leszno	Opole	Wrocław	Śnieżka
Jelenia Góra	0.85	0.90	0.89	0.87	0.90	0.82
Kłodzko		0.85	0.85	0.87	0.97	0.80
Legnica			0.94	0.90	0.94	0.81
Leszno				0.90	0.93	0.79
Opole					0.92	0.79
Wrocław						0.81

All *p* values lower than 0.01

**Table 3:** Average, minimum, maximum, and amplitude of UTCI values at 12 UTC for the years 1966–2017 in Lower Silesia

Stations	Jan	Feb	Mar	Apr	May	Jun	Jul	Aug	Sep	Oct	Nov	Dec	Year
Average (^∘^C UTCI)													
Jelenia Góra	− 10.8	− 8.9	− 5.5	2.4	11.3	15.3	18.0	18.5	12.6	5.6	− 3.7	− 9.4	3.8
Kłodzko	− 14.9	− 11.5	− 6.2	3.1	12.1	17.0	19.8	19.9	13.1	3.9	− 7.3	− 13.2	3.1
Legnica	− 14.9	− 12.3	− 6.6	2.9	12.3	16.2	19.5	19.6	12.7	4.5	− 6.2	− 13.0	3.0
Leszno	− 15.2	− 12.7	− 7.2	2.6	12.3	16.7	20.1	19.8	12.9	4.7	− 6.8	− 13.2	2.9
Opole	− 11.8	− 8.2	− 3.0	5.6	14.6	18.5	21.2	21.6	14.8	6.9	− 3.7	− 9.9	5.6
Wrocław	− 14.5	− 11.4	− 5.8	3.9	13.2	17.4	20.2	20.5	13.6	5.1	− 6.0	− 12.4	3.7
Śnieżka	− 43.7	− 42.0	− 36.0	− 26.0	− 14.7	− 9.5	− 6.3	− 4.2	− 13.9	− 23.6	− 35.9	− 42.1	− 24.7
Minimum (^∘^C UTCI)													
Jelenia Góra	− 48.1	− 47.5	− 40.5	− 33.7	− 23.7	− 19.9	− 11.0	− 15.6	− 13.2	− 30.7	− 42.4	− 46.9	− 48.1
Kłodzko	− 51.9	− 46.5	− 42.3	− 30.6	− 20.6	− 16.2	− 8.1	− 7.8	− 14.7	− 33.4	− 40.7	− 52.0	− 52.0
Legnica	− 46.9	− 44.2	− 43.8	− 33.7	− 21.6	− 10.5	− 11.4	− 8.4	− 18.7	− 27.3	− 43.0	− 43.8	− 46.9
Leszno	− 47.3	− 48.9	− 44.4	− 36.6	− 21.0	− 12.9	− 7.9	− 11.6	− 14.8	− 34.6	− 38.7	− 52.8	− 52.8
Opole	− 54.2	− 43.0	− 41.4	− 32.8	− 23.4	− 10.8	− 12.3	− 5.0	− 18.5	− 30.2	− 35.7	− 40.0	− 54.2
Wrocław	− 52.2	− 40.8	− 42.6	− 35.8	− 19.4	− 17.0	− 8.4	− 7.6	− 13.2	− 29.0	− 38.0	− 45.2	− 52.2
Śnieżka	− 72.8	− 70.0	− 70.0	− 63.4	− 55.7	− 51.4	− 44.5	− 46.7	− 52.9	− 60.8	− 66.5	− 72.0	− 72.8
Maximum (^∘^C UTCI)													
Jelenia Góra	19.0	22.7	28.0	32.6	34.6	36.2	40.3	38.5	36.8	30.4	21.8	16.9	40.3
Kłodzko	13.6	17.5	24.4	31.9	34.1	37.7	38.7	37.6	35.5	29.6	21.0	15.2	38.7
Legnica	15.7	18.6	24.2	33.3	34.2	38.1	40.2	40.6	37.3	31.8	19.8	16.1	40.6
Leszno	11.1	20.7	22.0	31.9	34.7	37.7	40.8	41.6	36.5	29.5	20.1	11.7	41.6
Opole	13.1	22.8	26.9	33.6	34.4	38.6	40.9	40.1	35.5	30.1	22.8	12.6	40.9
Wrocław	13.0	18.1	28.0	31.6	33.4	38.0	40.3	40.3	37.7	30.0	25.0	15.5	40.3
Śnieżka	10.1	12.7	17.4	18.0	20.5	26.4	29.8	27.0	23.1	23.2	16.3	12.0	29.8
Amplitude (^∘^C UTCI)													
Jelenia Góra	67.2	70.2	68.5	66.3	58.3	56.2	51.3	54.1	49.9	61.1	64.2	63.8	88.4
Kłodzko	65.5	64.0	66.8	62.4	54.7	53.9	46.8	45.4	50.2	63.0	61.6	67.2	90.6
Legnica	62.6	62.8	68.	67.0	55.9	48.5	51.5	49.0	55.9	59.1	62.8	59.8	87.6
Leszno	58.3	69.6	66.5	68.5	55.7	50.5	48.7	53.2	51.2	64.1	58.8	64.5	94.2
Opole	67.3	65.8	68.3	66.4	57.8	49.4	53.1	45.1	54.0	60.4	58.5	52.6	95.1
Wrocław	65.2	58.9	70.6	67.4	52.8	55.0	48.7	47.9	50.9	59.0	63.0	60.7	92.5
Śnieżka	82.9	82.7	87.4	81.4	76.2	77.8	74.3	73.7	75.9	84.0	82.7	84.0	103.0

### ARMA results

**Fig. 5:**
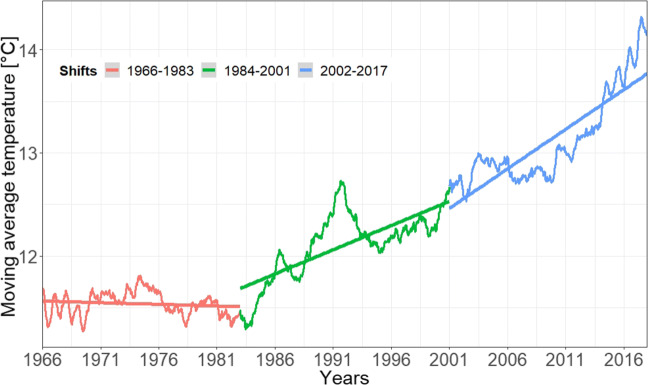
Eight years moving average of temperature in Wrocław for 1966–2017, with three linear trends for three homogeneous periods

The results of the ARMA model are presented only for the air temperature in Wrocław for the sake of clarity and synthesis. However, all model results are presented in Table 4. All time series were divided into three almost equally long periods: 1966–1983, 1984–2001, and 2002–2017 to which linear trends were fitted. As far as temperature at Wrocław station is concerned, goodness of fit returned *R*^2^= 0.197, *R*^2^= 0.552, and *R*^2^= 0.732 for the respective aforesaid time periods. The computed linear trends for each period and at each station were thus used to detrend the series (Fig. 5) (Maidment et al 1993).

**Table 4:** ARMA model proposal for the analysed period of 1966–2017 for all components and UTCI

Station	Parameter	Model	AIC	Model Par
Wrocław	Temperature	AR	0.187	*p* = 1; *q* = 0
	Cloudiness	ARMA	0.747	*p* = 2; *q* = 3
	Wind speed	−	−	−
	Vapour pres.	AR	0.451	*p* = 4; *q* = 0
	UTCI	AR	0.650	*p* = 4; *q* = 0
Kłodzko	Temperature	AR	0.294	*p* = 1; *q* = 0
	Cloudiness	ARMA	0.872	*p* = 4; *q* = 2
	Wind speed	−	−	−
	Vapour pres.	AR	0.452	*p* = 4; *q* = 0
	UTCI	AR	0.684	*p* = 3; *q* = 0
Jelenia Góra	Temperature	AR	0.310	*p* = 1; *q* = 0
	Cloudiness	AR	0.773	*p* = 4; *q* = 0
	Wind speed	−	−	−
	Vapour pres.	AR	0.478	*p* = 4; *q* = 0
	UTCI	ARMA	0.699	*p* = 2; *q* = 4
Legnica	Temperature	AR	0.304	*p* = 2; *q* = 0
	Cloudiness	−	−	−
	Wind speed	−	−	−
	Vapour pres.	AR	0.426	*p* = 3; *q* = 0
	UTCI	AR	0.674	*p* = 4; *q* = 0
Leszno	Temperature	AR	0.280	*p* = 2; *q* = 0
	Cloudiness	−	−	−
	Wind speed	−	−	−
	Vapour pres.	AR	0.466	*p* = 4; *q* = 0
	UTCI	AR	0.636	*p* = 4; *q* = 0
Opole	Temperature	AR	0.287	*p* = 1; *q* = 0
	Cloudiness	ARMA	0.870	*p* = 3; *q* = 4
	Wind speed	−	−	−
	Vapour pres.	AR	0.454	*p* = 2; *q* = 0
	UTCI	AR	0.685	*p* = 3; *q* = 0
Śnieżka	Temperature	AR	0.302	*p* = 2; *q* = 0
	Cloudiness	ARMA	0.740	*p* = 2; *q* = 3
	Wind speed	−	−	−
	Vapour pres.	AR	0.542	*p* = 2; *q* = 0
	UTCI	ARMA	0.779	*p* = 3; *q* = 2

Missing models of cloudiness are due to gaps in the missing data which did not allow for a stochastic model

**Fig. 6:**
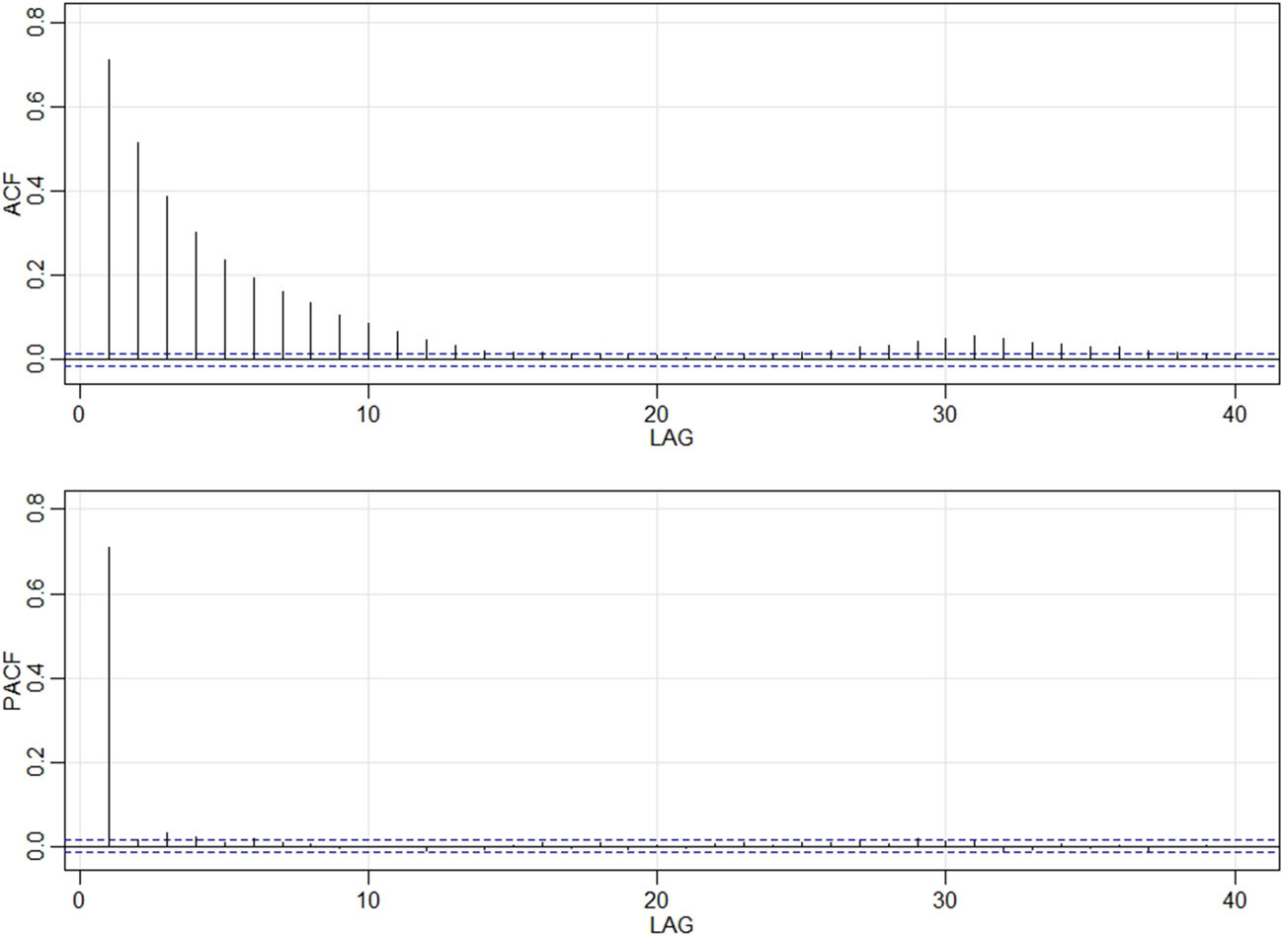
Autocorrelation and partial autocorrelation functions of detrended and standardised signal of air temperature in Wrocław; the discontinuous blue line indicates the confidence interval below which autocorrelation is statistically insignificant

The next step in the decomposition process was standardisation, which we obtained by subtracting the daily mean, here assumed to be equal to the monthly one, and then dividing it by the standard deviation of the corresponding month. In doing so, we assumed that daily changes are negligible compared with monthly ones, and the resulting process also allowed removing the effect of seasonality. After standardisation, we reasonably assumed the statistical stationarity and homogeneity within the three time periods and then computed both ACF and PACF for the whole length of the series. Both ACF and PACF are shown in (Fig. 6).

**Fig. 7:**
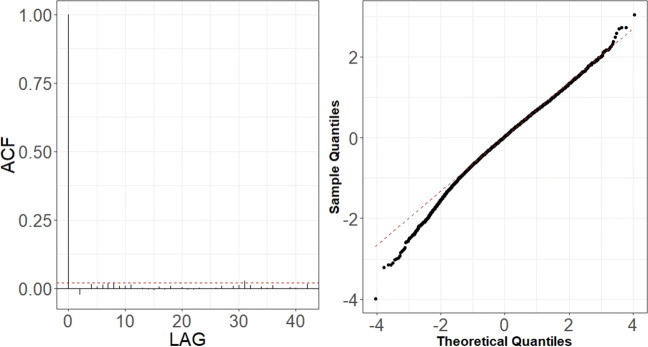
Example of ACF residuals (red line, level of confidence interval) of AR(1) model for air temperature in Wrocław (left) and comparison with theoretical quantiles, the red dashed line represents the ideal distribution of data (right)

**Fig. 8:**
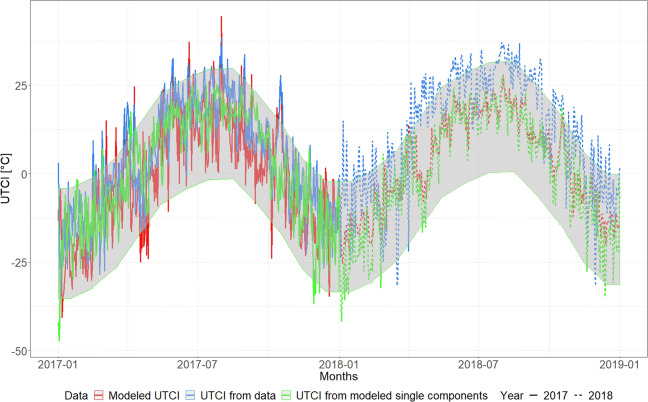
Reconstruction (continuous line) and forecast (dotted line) of the UTCI compared with the measured series with *σ*^1^ range (grey area) using Wrocław as example

ACF tails off gradually over several lags, where as PACF becomes 0 almost immediately after lag 1. This suggests that an autoregressive model order 1, namely AR(1), could be sufficient to remove all correlations in the signal thereby obtaining the uncorrelated residuals and good correspondence between the modelled and sample data (Fig. 7). For a specific station, the AR(1) scored an AICc value of 0.1871, which suggests a good performing model (Sakamoto et al. 1986). The present model can also be made to generate new data, which must be de-standardised and summed according to the original monthly mean and linear trend in order to obtain the statistically equivalent time series (Fig. 8) to the real data. It is important to appreciate how the predicted temperatures (in red) are all contained within the grey belt, which has a width that is equal to ± 1 standard deviation (*σ*^1^). The *σ*^1^ according to so-called three-sigma rule of thumb statistically represent at least 70% of the measured data (Wheeler et al. 1992). Thus, the red signal is clearly just one possible realisation of the stochastic process (Fig. 8).

Whilst the proposed linear stochastic approach was shown to be successful in decomposing all time series into deterministic and noise components, this was not the case for the wind speed. For this variable (Table 4), the decomposition of the time series did not lead to satisfactory results for any ARMA model order that we tested. This may have been caused by the vectorial origin of the variable itself, where changes in wind direction with similar magnitude might have produced ambiguity in the data. This generates some spurious temporal correlation, which cannot simply be removed by linear autoregressive models. The UTCI was then computed from the data (blue line in Fig. 8) with the purpose of comparing it with its stochastic model, or with the one obtained from single stochastic models for each meteorological component. Figure 8 shows that fitting a stochastic model on the UTCI after applying the decomposition steps already described produces a stochastic model that successfully removes all correlations and separates the noisy component from the correlated one (green line). This model was built using all UTCI data up to 2017 and then using 2018 data as a prediction.

**Fig. 9:**
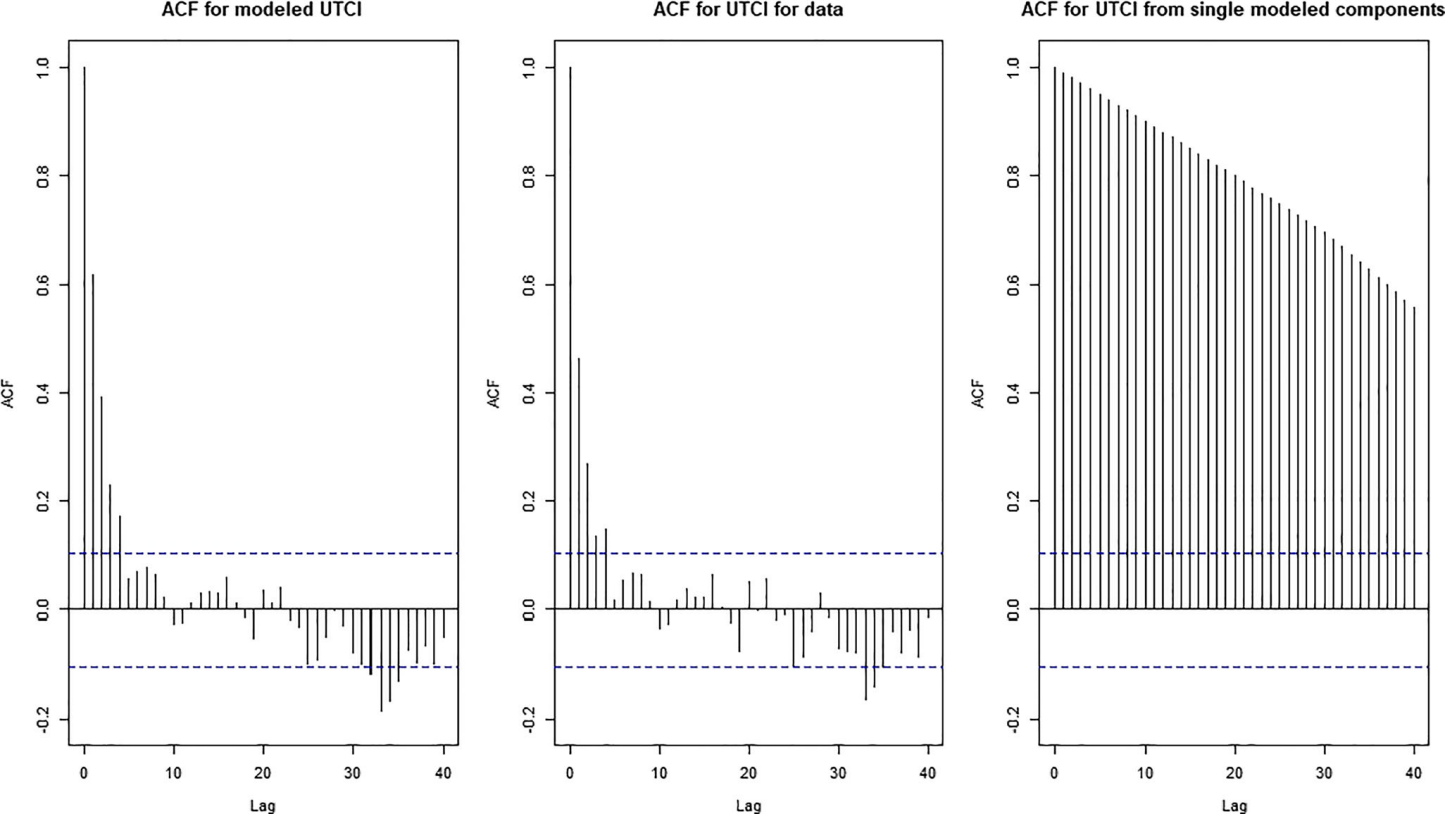
Autocorrelation functions for 2 approaches of modeled UTCI compared with the UTCI calculated from the data using Wrocław as example

We also tried to compute the UTCI starting from the stochastic models of each single meteorological component including the wind speed, of which not all correlations could successfully be removed as already described. This resulted in a fluctuating signal (red line in Fig. 8) very similar to the green one in terms of visual appearance in the time domain. This would also result in similar probability density distributions, even though the autocorrelation function might be substantially different (Fig. 9).

## Discussion

The average annual UTCI values in the Lower Silesia region range from 2.9 ^∘^C in Leszno to 5.6 ^∘^C in Opole (Table 3). Slightly higher values can be found in the literature. Rozbicka and Rozbicki (2018) showed that the annual average ranged from 3.5 to 9.3 ^∘^C for the period 1998–2015. According to Mękosza (2013), the average UTCI values in the Lubuskie province change between 6.1 ^∘^C in Zielona Góra and Gorzów Wielkopolski and 8.1 ^∘^C in Słubice. Błażejczyk et al. (2014) calculated an annual UTCI value of 4.1 ^∘^C in Warsaw and 5.7 ^∘^C in Kołobrzeg, for the period 1991–2000. However, those studies were carried out over shorter periods and in more recent years, where the influence of ongoing climatic changes may have affected the result, thus explaining the slightly higher values. This notwithstanding, all studies correspond to the same heat stress class of “slight cold stress.” The situation for Śnieżka is significantly different, it being a mountain station, where cold stress at low temperatures is considerably increased by the high wind speeds (Bröde et al. 2012a). The Śnieżka Mountain has the most commonly used hiking trails in Karkonosze and knowledge of the local bioclimate can be useful for supporting future tourism in the area. Due to frequent high wind speeds, the average UTCI value is as low as − 24.7 ^∘^C. It is worth stressing that cutting off the wind speed to 20 m⋅ s^− 1^ (Novak 2013), which we imposed in order for the UTCI formula to be applicable, has not affected the interpretation of UTCI in terms of thermal comfort. For those regions, the limiting wind speed of 20 m⋅ s^− 1^ already represents high cold stress classes that require additional protective measures, especially from the wind (Błażejczyk 2011a). This is an important piece of information especially for tourism applications, particularly if such a combination of meteorological events leading to extreme cold happens during the spring and summer seasons. Most of the time, tourists might be unprepared for exposure to such extremes (e.g. as occurred in Śnieżka in May where for the analysed period, 30% of the thermal conditions corresponded to strong cold classes (Fig. 2). These findings may also have applications in analysing the bioclimate in arctic windy conditions (Araźny et al. 2019). Each station shows a weak upward trend for UTCI, which is in line with the air temperature and solar radiation trends that occur in Wrocław for the analysed period (Bryś 2010, 2013). All synoptic stations also show UTCI seasonal patterns that reflect the Polish annual climate (Marsz et al. 2016, 2019). Two heat waves were recorded for the “very strong heat stress” class in 1994 and in 2015. For example, for Opole in 1994, the heat wave persisted for 6 days, i.e. from July 28 to August 2. In Wrocław, Leszno, and Legnica, the heat wave was also classified as “very strong heat stress” and lasted 4 days, i.e. from July 29 to August 1. In Jelenia Góra and Kłodzko, values were 1–2 ^∘^C UTCI below the “very strong heat stress” class threshold. In 2015, a strong heat wave lasted from of August 7–12 in Opole, while it was cooler by 5–6 ^∘^C of UTCI in other cities. In both heat waves, Jelenia Góra and Kłodzko had the lowest values of UTCI. The 2015 heat wave was also presented for Warsaw by Rozbicka and Rozbicki (2018) where the period August 3–16 was analysed. The number of “strong heat stress” class events for all stations in the analysed period was 1452, which corresponds to a relative frequency of about 1%. Therefore, events leading to heat stress are rather rare and we can assume that in the studied area there is low risk of thermal comfort disruption caused by thermal classes of the warm type (Bröde et al. 2012a). As far as cold thermal classes are concerned, there were 5613 observations of “extreme cold stress” in Śnieżka (i.e. 29% of the time), and 1 observation in Leszno and Legnica in 1996 (0.005%). “Very strong cold stress” occurred 7288 times (5.5% of all station observations) in 5406 days over 52 years. In terms of individual stations, 457 times (2.5%) occurred in Jelenia Góra, 722 times (4%) in Kłodzko, and 664 times (3.5%) in Legnica and Leszno, 661 times (3.5%) in Opole, 339 times (1.5%) in Wrocław, and 3878 times (20%) in Śnieżka. In summary, “extreme cold stress” represents 4.3% of all observations (mainly in Śnieżka), “very strong cold stress” 4.8%, “strong cold stress” 14.8%, and “moderate cold stress” 22.6%. The “slight cold stress” class occurred with a frequency of 17.4%; “no thermal stress” 29.7%, which was the most common thermal class for the analysed period and area, “moderate heat stress” 4.9%, and “strong heat stress” and “very strong heat stress” 1.5% (Fig. 3). These results are similar to those registered in other parts of Poland (Kuchcik et al. 2013; Błażejczyk et al. 2014; Rozbicka and Rozbicki 2018).

We performed a stochastic modelling of the meteorological variables and of the UTCI, using the results for air temperature in Wrocław station as an example. Removing the trends (Fig. 5) and seasonality was necessary in order to use the ARMA models (Fig. 7). The ARMA models were estimated according to the sample auto- and partial- correlation functions (Fig. 6). All models were successful in removing all correlations from noise for all components except for wind speed (Table 4), as shown for example by the comparison with theoretical quantiles for the temperature signal (Fig. 7). As shown in Fig. 8, the prediction underestimates the actual air temperature which is the result of a very warm year in 2018 compared with the past 52 years. The modelled UTCI signal correctly oscillates within the ± *σ*^1^ variability band as the calculated one (blue line) up to the year 2017 (Fig. 8). However, the model also appears to return lower values for 2018 according to the results described for the temperature above. The higher statistical variability for the year 2018 could not be captured by the stochastic model fitted with only the variability of previous years given the ergodicity assumption preserved by the model. It is also worth recalling here that the linear properties of a fluctuating time series are completely identified not only by its probability density function, but also by its temporal correlation. Indeed, this is precisely the property that escapes from a comparison of the UTCI computed from the individually modelled components and the UTCI directly computed from data or from its stochastic model (Fig. 9). Although the magnitude of the variability might be the same for the two modelling approaches, the autocorrelation function of the UTCI reconstructed from the stochastic modelling of each single variable (Fig. 9, right-hand panel) shows a completely different decaying tail, suggesting “too long” a correlation compared with the UTCI obtained from the data (Fig. 9, central panel) and that from its direct stochastic modelling (Fig. 9, left-hand panel). This phenomenon might well be the result of the way the residual correlation in wind speed propagates and is amplified by the UTCI formula, which uses monomial power law relationships of up to the 6th degree for wind speed and its multiplication with other variables.

The presented approach based on stochastic models that have found broad applications in hydrology (Maidment et al 1993) and the economy (Milani et al. 2017) is therefore appealing for use in bioclimatology as well. However, autoregressive models have successfully recognised and decomposed linear properties (i.e. temporal autocorrelation) of the time series. Somehow counterintuitively, our results suggest that in order to predict UTCI or generate statistically equivalent data, a direct autoregressive model of the UTCI would be more successful than starting from individual models for the single components that make up the UTCI. As all analysed stations are also spatially correlated (Table 2), stochastic models allow for generating statistically equivalent dataset that can be used to reconstruct the bioclimatic conditions in the region.

## Conclusions

This study analysed the bioclimatic conditions of Lower Silesia (SW Poland) for the period 1966–2017. A long-term analysis of the bioclimate of Lower Silesia showed many similarities to other parts of Poland (Kuchcik et al. 2013; Mękosza 2013; Błażejczyk et al. 2014; Rozbicka and Rozbicki 2018). The most favourable biometeorological conditions occur between April and October. The most common thermal class is “no thermal stress,” which is also consistent with cases found in the literature. In the case of windy conditions as in Śnieżka located at the top of the mountain, calculation of the UTCI requires that wind speed be cut off at its uppermost limiting value. Although this may well be questionable as it introduces a strong nonlinearity and discontinuity in the resulting time series, its final effect on UTCI may still be correct if the index falls into those classes representing extreme stress conditions. This was the case for the stations analysed in this study, even though we would still recommend that this forced approach should be verified on a case-by-case basis. Forecasts for the air temperature and UTCI for Wrocław were presented using a comparison of the measured data with those of the corresponding stochastic models. However, the presented ARMA modelling approach has shown itself not to be applicable for wind speed, specifically in successfully removing all temporal correlations from the residuals. Accordingly, the UTCI calculated from the single modelled components failed to reproduce some of the linear statistical properties of the real signal, that is, its temporal autocorrelation. On the contrary, the autocorrelation model of the UTCI signal seemed successful and can thus be used for reconstruction and forecasting purposes. This work paves the way for future studies that aim, for instance, not just at clarifying the propagation of the temporal correlation across the formula for the UTCI, but also at reconstructing the dynamical system governing regional bioclimatic conditions.

## Electronic supplementary material

Below is the link to the electronic supplementary material.

(RAR 4.05 MB)
